# Stress and defense responses in plant secondary metabolites production

**DOI:** 10.1186/s40659-019-0246-3

**Published:** 2019-07-29

**Authors:** Tasiu Isah

**Affiliations:** Department of Botany, School of Chemical and Life Sciences, Hamdard University, New Delhi, 110 062 India

**Keywords:** Plant physiology, Phytochemistry, Biotic and abiotic stress, Antioxidants, Phytochemicals, Plant secondary metabolites, Natural products, Plant nutrition, Oxidative stress

## Abstract

In the growth condition(s) of plants, numerous secondary metabolites (SMs) are produced by them to serve variety of cellular functions essential for physiological processes, and recent increasing evidences have implicated stress and defense response signaling in their production. The type and concentration(s) of secondary molecule(s) produced by a plant are determined by the species, genotype, physiology, developmental stage and environmental factors during growth. This suggests the physiological adaptive responses employed by various plant taxonomic groups in coping with the stress and defensive stimuli. The past recent decades had witnessed renewed interest to study abiotic factors that influence secondary metabolism during in vitro and in vivo growth of plants. Application of molecular biology tools and techniques are facilitating understanding the signaling processes and pathways involved in the SMs production at subcellular, cellular, organ and whole plant systems during in vivo and in vitro growth, with application in metabolic engineering of biosynthetic pathways intermediates.

## Background

Because they are sessile organisms, plants have evolved numerous mechanisms for accommodating changes arising in their fluctuating growth conditions to enable functional flexibility under the influence of environmental factors without affecting cellular and developmental physiological processes [1, 2] by producing repertoire of secondary metabolites (SMs) that play variety of roles in response to changing environment, growth and development [3, 4]. The changes may be induced by environmental components that include local geo-climatic and seasonal changes, external conditions of temperature, light, humidity and developmental processes, among others, and impact biomass production and biosynthesis of plant secondary metabolites (PSMs) [4–8]. The secondary molecules are produced occasionally in living plant cells and do not play much of significant role in the primary life of plants that produce them, with the production been at low concentration commensurate with growth physiology of a plant species [9]. Production of the metabolites by the plants is regarded an adaptive capacity in coping with stressful constraints during challenging and changing environment of growth that may involve production of complex chemical types and interactions in the structural and functional stabilization through signaling processes and pathways [10]. Vast number of the secondary molecules are biosynthesized from primary metabolites and accumulated in plant cells, and the production could be induced in the in vitro condition when cell cultures are treated with biotic, abiotic elicitors and signaling molecules [6, 11–13]. For centuries, humanity has exploited such physiological adjustments in plants as a source of improving biosynthesis of bioactive compounds they produce which are useful in making drugs or direct use of the plants as herbal medicine to cure diseases and ailments, and still, about 25% of the drugs in use by the humanity are derived from medicinal plants [14, 15]. However, more than 350,000 species are yet to be investigated on production of the biopharmaceuticals.

In the past recent decades, studies on bioactive compounds produced by the plants have shown their nutritional value in the form of flavors, food addition and as biochemicals having industrial use [16–19]. Their production by a plant species is dependent on growth condition and physiology, and to great extent due to the differential impact of environmental growth conditions on metabolic pathways associated with their biosynthesis. Many of the reported studies have shown variety of ecological functions ascribed to the plant secondary metabolism (PSM), from protection against environmental stresses to defense during pathogen, insects, and herbivores attack [20–22]. This lead to the use of considerable number of elicitors of biotic and abiotic origin in improving production of the metabolites in plant cells through the use of in vivo and in vitro growth conditions manipulation, and increasing evidences from results of the studies have established the role of oxidative stress defense response in production of the PSMs [22–27]. Further, antioxidant and anti-radical functions have been ascribed to their production in plants, so as to assist them in coping with oxidative stress situations during unfavorable growth conditions of the in vivo and in vitro that may involve participation of hydroxyl or thiol group-containing compounds. This may involve production of chemitypes that includes lipoic and ascorbic acid, o-dihydroxy group-containing flavonoids like carotenoids, arylamines, quercetin, aliphatic and unsaturated fatty acids among others [10, 28, 29]. In the past recent decades, studies have also shown that exposure of plant cells to a stressful growth condition(s) may result in an exchange between carbons to biomass production for the biosynthesis of defensive plant secondary compounds [30] when the stress situation is adequately recognized [6, 10, 13]. Recent evidences have shown that the cellular stress response communications associated with biosynthesis of the PSMs involve extensive cross-talk and signaling processes between pathways in plant cells that may involve participation of molecules such as salicylic and jasmonic acids, calcium, abscisic acid, polyamines and nitric oxides [6, 10, 11, 31]. However, the chemical rationale of the signal transduction system involved is yet unclear. Stress physiology related to the PSM have continued to receive considerable attention in the recent, and increasing evidences from the many literature reports suggests stabilizing role of the metabolites in plant cell structures during in vivo and in vitro stress growth conditions [6, 11, 27, 32, 33]. Many of the studies applied metabolomics and transcriptomic technologies in investigating and understanding stress-associated genes and pathways involved in biosynthesis of the PSMs in medicinal plants [11, 34, 35]. This in many instances involved application of improved bioinformatics pipelines augmented by increased sequenced genomes of many of the plants and secondary compounds they produce to aid understanding complex processes associated with the biosynthesis through application of many approaches in elucidating spatial and temporal production of the bioactive compounds in relations to developmental processes and environmental regulation [34–38]. Data generated from the metabolomics and genomic studies along with efficient use of the technologies in biosynthetic pathways enzymes characterization have facilitated understanding processes involved in the production of many PSMs in the recent [34–36, 39]. For instance, application of organic synthesis coupled with the technologies have aided characterization of complex processes involved in the biosynthetic pathway(s) of taxol [36], carnosic acid and forskolin [39–43] among others. However, analytical limitations that include reliable identification and quantification, the metabolomics size of the plants and tissue-specific variation [35] in their production still pose considerable challenge. The available substantial evidence(s) have indicated antioxidant and anti-radical functions been played by the PSMs when plants are coping with oxidative stress situation during unfavorable growth conditions of the in vivo and in vitro [44, 45]. However, there are still difficulties in ascertaining the stress physiology and metabolic effect of their production to specific stress factor, given the variety of simultaneous and interconnected effects of complex stress factors to metabolic processes in plants, and opposing signaling responses in distinct pathways that are involved in defense response function associated with production of the SMs in plant cells can be implicated [11, 22, 26, 27]. As a result, the approach of “carbon-based secondary metabolites” and “source-sink carbon-nutrients balance” which are on the premise that stress that suppresses growth more than photosynthesis promotes accumulation of the SMs hypotheses are current models used to predict their production under stressed conditions [46, 47]. It is believed that higher production of most of the metabolites by the plants is part of chemical defense response system associated with increased resistance to stress, and possible explanation to existence of variation in defense response(s) employed by various plant taxonomic groups, as reflected by the type and concentration of the secondary metabolites (SMs) they produce [20, 22, 26, 48, 49] given that a secondary metabolite (SM) can perform specific stress response function in plant cells [11, 50–53]. For instance, production of sesquiterpenes is associated with defense response system in members of the family Solanaceae, glucosinolates-myrosinase are produced by the Brassicaceae members, stilbenes with the Vitaceae, isoflavones with Fabaceae while limonoids are produced by Rutaceae and Meliaceae [21, 26]. Hence, the possible reason production of the PSMs is at low and varied level, and to great extent, dependent on the physiological and developmental stage or process in most plants. As a result, biotechnological approach of in vitro technology through strategies and approaches have found application in their production [38, 54–56]. Many of the efforts put together have resulted in the achievement(s) of higher yield in the employed production systems (Table 1) with culture medium manipulation the most employed approach in the recent decades [38]. This review summarizes recent trends in reported data on the physiology of PSMs production across plant species, cultivars, and genotypes with emphasis on relationship to biosynthesis of the molecules.

**Table 1:** Some of the secondary metabolites produced in the plant cell, tissue and organ in vitro cultures

Secondary metabolite	Plant source(s)	In vitro production	Medicinal use(s)	Reference(s)
Artemisinin	*Artemesia annua*	C, SH, SE, ET, THR, UTHR	Anti-malarial	[296]
Camptothecin	*Camptotheca acuminata, Nothapodytes nimmoniana*, many members of the family Icacinaceae, and many other plant species from unrelated families	C, SH, SE, THR, UTHR	Anti-cancer	[297–303]
Codeine	*Papaver somniferum*	C, SH, SE, UTHR	Sedative	[304–309]
Robustaquinones	*Cinchona robusta*	C	Anti-malarial, numerous bioactivities	[310–313]
Securinine alkaloids	*Securinega suffruticosa*	C, SH	Cytotoxicity, anti-alzheimer and many bioactivities	[314–316]
Ajmaline	*Rauvolfia serpentina*	C, SH, SE, THR, UTHR	Anti-hypertension	[317, 318]
Diosgenin	*Dioscorea deltoidea*	C, SH, SE, THR, UTHR	Steroidal precursor	[319–324]
l-Ephedrine	*Ephedra sinica, Ephedra* genus members	C, SH, S, UTHR	Diatary supplement	[325–330]
Ellipticine	*Orchrosia elliptica*	C, SH	Anti-cancer	[331–333]
Bacosides	*Bacopa monnieri*	C, SH, THR	Neuroprotective and many bioactivities	[334–339]
Altamisine	*Ambrosia tenuifolia*	C, SH	Numerous bioactivities	[340]
Matrines	*Sophora* species	C, SH	Anti-cancer, many bioactivities	[341]
Rosmarinic acid	*Salvia miltiorrhiza*	C, SH, SE, ET, THR, UTHR	Anti-oxidant, anti-microbial	[342]
Rohitukine	*Dysoxylum binectariferum*	C, EN	Anti-cancer, numerous bioactivities	[343, 344]
Stevioside	*Stevia rebaudiana*	C, SH, SE	Sweetener	[345–349]

### Stress and defense responses in relations to the production of plant secondary metabolites

Stress response in plants comprises repertoire of molecular, cellular cross-talk and signaling responses initiated through the detection of specific or combined biotic or abiotic stress effect that may result in the induction of SM [58]. Plants immune system have evolved numerous stress detection mechanisms that includes transmembrane recognition (in response to evolving pathogen or microbial association molecular pattern), polymorphic NB-LRR protein production by most R-genes (to large extent inside cell) and production of SMs to cope with the stress situations, and thus, become remodeled to endure the condition [59, 60]. This may be achieved through influence on physiological processes in plant cells, triggered by signal transduction process to accommodate the stimulus involving adjustments in primary and SM that enable regulation of cell osmotic pressure, prevents cell components oxidation, pathogenic microbial growth and infection, and deter herbivores [11, 31, 60] through biochemical and physiological processes associated pathways regulation. Induction of the stress may stimulate expression or repression of stress-genes network through precise regulation that may result in the production of functional cellular molecules to accommodate the stress effect [61–63] which may be in the form of biosynthesis of osmoprotectants, detoxification enzymes, transporters, chaperones and proteases that serve as the first line of cellular protection [64]. In many instances, also, the stress response involves reversible salicylic and jasmonic acid production, ethylene and reactive oxygen species (ROS) production, ion fluxes, phosphorylation, promoter elements and transcription factors [65]. Recent evidences have shown the role of regulatory proteins activation and signaling molecules in regulating signal transduction processes and expression of stress-responsive genes as an early response that prevents cellular damage and re-establish homeostatic state essential for growth of plants during in vitro and in vivo stress growth conditions [11, 64, 66–68]. However, knowledge about the effect of stress on the PSM is to a large extent, based on research efforts towards yield maximization of bioactive constituents from herbs, spices, and medicinal plants through evaluating tissue or organ physiology while understanding cellular functional role the metabolites are playing in plant cells is stimulating interests, particularly on the production during in vivo and in vitro growth. On the other hand, defense response system involves production of an array of chemical, structural and protein-based physio-molecular response(s) against invading foreign body or organism into plantsˈ systems through variety of responses found in various plant taxonomic groups [20, 22, 26, 69]. The defense response system becomes activated when intra- or extracellular signal is received by receptors in cell plasma membrane, involving their binding accompanied by signal transduction cascade initiation that may result in de novo synthesis or activation of transcription factors responsible for regulating SMs biosynthesis genes expression [23]. This may lead to systemic adjustment and, in many instances, a diseased condition or even becomes regular part of physiological processes [21, 31]. Of importance in the defense response system is the perception of the stress that leads to initiation of efficient recognition and basal defensive mechanism for activation of different signaling cascades associated with a given stress effect [31, 70, 71]; defensive response may be constitutive or induced with the former and its secondary compounds always present in plant, and often species-specific in existence in the form of stored compounds, precursors of active compounds that may be easily activated in response to damage caused on plant body or conjugated compounds [21, 72]. The latter gets initiated after the actual damage occurs on plant body and may involve production of defensive proteins that includes lectins and protease inhibitor(s) or production of toxic SMs [21]. Recent understandings suggest that induction of a defense response system may involve wounding and recognition of elicitor compounds that could lead to trigger of signaling pathways and resultant initiation of action at distant region of plant [26]. For instance phytoalexins are produced by many plant species in response to microbial invasion to serve defensive response function. Similarly, production of isoflavonoid phytoalexins in soybean and alfalfa and sesquiterpenes by members of the family Solanaceae is another example of their defensive function. Overall, both stress and defense response processes stimulate metabolic changes that may result in the biosynthesis of bioactive compounds having pharmaceutical or nutritional value.

Plant secondary metabolites could be detected in cells of the whole plant body but, site of biosynthesis, in most of the cases, is restricted to an organ and transported to different region through vascular tissues or symplastic and apoplastic transport to the site of storage, depending on polarity of the metabolite [35, 73]. Hydrophilic compounds that include alkaloids, glucosinolates and tannins are stored in vacuoles or idioblasts whereas lipophilic such as terpene-based essential oils could be stored in thylakoid membranes or cuticles, resin ducts and trichomes [73, 74]. The sites or storage tissues and structures may include leaves, shoots, roots, flowers, callus or somatic embryos and specialized accumulation sites such as glandular trichomes, periderms, and phellem among others. For instance, monoterpenes produced by members of Labiatae are biosynthesized in secretory cells but, become accumulated in epicuticular cavity of glandular trichomes [75]. In the past recent decades, it has been established that spatial and temporal change in function related to production of the PSMs in many storage sites could be encountered, based on the growth physiology and developmental stage of plant species investigated [35, 51–53, 76–79]. Thus, accumulation of a SM in plant at higher levels could be an indicator of high expression of genes and metabolic pathway for its biosynthesis in cells, although translocation of a bioactive compound from site of biosynthesis to storage site plays significant role with some of the PSMs [1, 80]. For instance, involvement of membrane transportation system through ATP-binding cassette (ABC) transport has been implicated in the accumulation of PSMs in many medicinal plants [81]. In a study aimed at understanding the kinetics of berberine production and storage in the cell cultures of *Coptis japonica*, Sato et al. [82] demonstrated concentration gradient-based uptake of the alkaloid when added into the culture medium of cultured cells of the species, and its subsequent accumulation in vacuole of cells. Transport of the alkaloid involved uptake at the levels of plasma membrane and subsequent efflux of berberine in cytosol and into vacoular lumen at tonoplast levels. Recent evidences on production of some PSMs to specific structures in plant body have also implicated their protective functional role through defense response in the growth environment [26, 73]. Of worthy note is the fact that much of the above information and understandings about the role of stress and defense responses in PSMs production either involves application of plant cell culture or pot experiments [83–85], with medicinal plant *Catharanthus roseus* as the model species widely investigated for production of its anticancer alkaloid vincristine and vinblastine [86]. Over three decades ago, Wink [87] suggested that production and concentration of a SMs produced by a plant species is determined by equilibrium relationship between biosynthesis, storage, and degradation, based on the stage of development as to which becomes dominant. In many recent literature reports, it had indeed been shown that an array of responses involving signal transduction systems and molecules with influence on tight regulation of biosynthetic pathway(s) could be characterized in different plant species, genotype or cultivars due to ecotype and genetic component-dependent variations response to the stress or defense response function during growth condition(s) of plants. This influences SM, depending on the season, environmental or external triggers [8, 22, 26, 73]. For example, production of essential oils in the trichome of leaves imply their defensive role against herbivores or insect predators while tannins production in the vacuoles of leaves cells located beneath epidermal surface and their bitter taste deter predators [73, 88]. Over 3 decades ago, Wink [87] also opined that higher accumulation of alkaloids in the seeds of most plants could be considered a chemical defensive strategy, and for use as source of nitrogen during germination. Competition with microbes and other essential mineral nutrients, defensive pathogens and herbivores attack to plants may induce SM pathways in plant cells [21, 50]. This may involve hypersensitive response that leads to localizing an invading pathogen by plant system at the infection site, with phenolic-storing cells playing vital role in programmed cell death [89, 90]. Serotonin reported from many biota is believed to be involved in various physiological processes in many plants through protection from environmental stresses and against pathogenic invasion, as well as a role in scavenging ROS that leads to delayed senescence [6, 91]. It was reported to serve protective function from environmental stress in the reproductive tissues of young *Datura metel* through antioxidant role, and exposure of flowers to cold stress significantly enhanced the production [92]. Polyamines, spermidine, spermine and putrescine found in wide range of biota are involved in variety of physiological processes that include senescence, development and stress responses [93]. Production of the polyamines at higher cellular levels by plants is associated with tolerance to environmental stresses. Thus, stress-tolerant plants possess high capacity for their enhanced biosynthesis during abiotic stress growth conditions, and certain polyamines could act as elicitors to the production of PSMs [93]. Xanthophyll that contains conjugated double bonds in their long chain is involved in xanthophyll cycle, and performs the function of excess light dissipation into harmless heat energy in plant cells [94] while phenolics storing cells play a vital role in the development of programmed cell death [90]. Although the exogenous application of jasmonates to the plants had been proven to cause morphological and physiological effects, they are also associated with the production of PSMs that form an integral part of the defense responses [95]. Their application stimulated biosynthesis of many SMs in the cell cultures and intact plant species [95]. Flavonoids, phenolics and polyphenolics are ascribed significant role in plant antioxidant responses and development, pigment and lignin biosynthesis [96]. The above examples have shown few among the enormous SMs and signaling molecules produced by plants in response to the stress or defense signal function, and variation within genotype, taxonomic group and physiology, and experimental technologies employed in their evaluation is variable. Similarly, the metabolites perform varied physiological cellular functions essential for growth of the plants at varied degree. However, it is still difficult to ascertain their stress and defensive function given the poor understanding of cellular level functions and spatial and temporal changes encountered in the production, based on experimental approach employed. Additionally, demarcation on the production of a specific metabolites in response to the stress and defense response function is difficult, and as such, ascertaining the existence of interconnection between primary and secondary metabolic pathways that provide precursors to the SM pathways in plant cells is difficult. In many recent studies, it had been shown that SM system in plants is a response to the stress and defensive situations that leads to an enhanced biosynthesis of the metabolites in an integrated defense mechanism through dynamic ways (e.g. Tables 1 and 2). However understanding the signaling processes involved and their interconnection with the primary metabolism is yet unclear, and very few had been investigated in some taxonomic groups, based on plant tissues or organs evaluated with rare reports on whole plant system evaluation or cellular levels.

**Table 2:** Production of some plant secondary metabolites under various in vivo growth condition of plants

Secondary metabolite	Plant source(s)	Tissue analyzed	Growth condition	Reference(s)
Artemisinin	*Artemesia annua*	Whole seedling (treated and control)	Salt, drought and water logging	[350]
Camptothecin	*Camptotheca acuminata*	Seedlings	Nitrogen, drought and anti-transpiration agents	[125, 128]
Codeine	*Papaver somniferum*	Plantlets	Drought stress	[260]
Rosmarinic acid	*Salvia miltiorrhiza*	Leaves, roots and aclimatized plantlet shoots	Hydroponic culture	[351]
Rohitukine	*Dysoxylum binectariferum*	Seedling (roots, collar region of stem and young leaves)	Normal	[352]
Stevioside	*Stevia rebaudiana*	Leaves (dried)	Hydroponic culture, salt stress	[353–355]
Allicin	*Allium sativum*	Whole plant	Pot experiment on light effect	[356]
Andrographolide	*Andrographis paniculata*	Leaves and stem	Open field experiment with plant populations	[357]
Resveratrol	Grapes, Groundnut	Leaves, shoot, roots and whole plant	Numerous	[358, 359]
Betalain pigments	Caryophyllales members	Different plant parts	Different growth condition	[360]
Saikosaponins	*Bupleurum chinense*	1-year-old plants, plants	Drought, watering and re-watering, fertilization	[294, 361]
Hyoscyamine and scopolamine	*Atropa belladonna* extracts	32-week-old dried root	Irrigation in greenhouse experiment	[362]
Capsaicin	*Capsicum* sp.	Fruits	Salinity-induced stress	[363]
Sennosides	*Cassia augustifolia*	Pre-, post and flowering plants	Pot culture experiment	[364]
Indole alkaloids	*Catharanthus roseous*	Leaves	Greenhouse under binary stress-induced condition	[365]
Asiaticoside and madecassoside	*Centella asiatica*	Leaves (post-harvest)	Low temperature and water dehydration	[366]
Valepotriates	*Valeria* species	All organs	Normal growth condition (Iran)	[367]
Rutin	*Dimorphandra mollis*	All plant parts at different growth stages	Normal, drought, flooding and salinity	[368]
Furanocoumarins	*Bituminaria bituminosa*	Leaves dry matter and fruits	Field conditions and hydroponics	[369]
Glycyrrhyzin	*Glycyrrhyza glabra*	Plants at seedling and adult stage, stolons	Drought stress	[370]
Zealexins and kauralexins	Maize	Roots	Drought stress	[371]

**Fig. 1:**
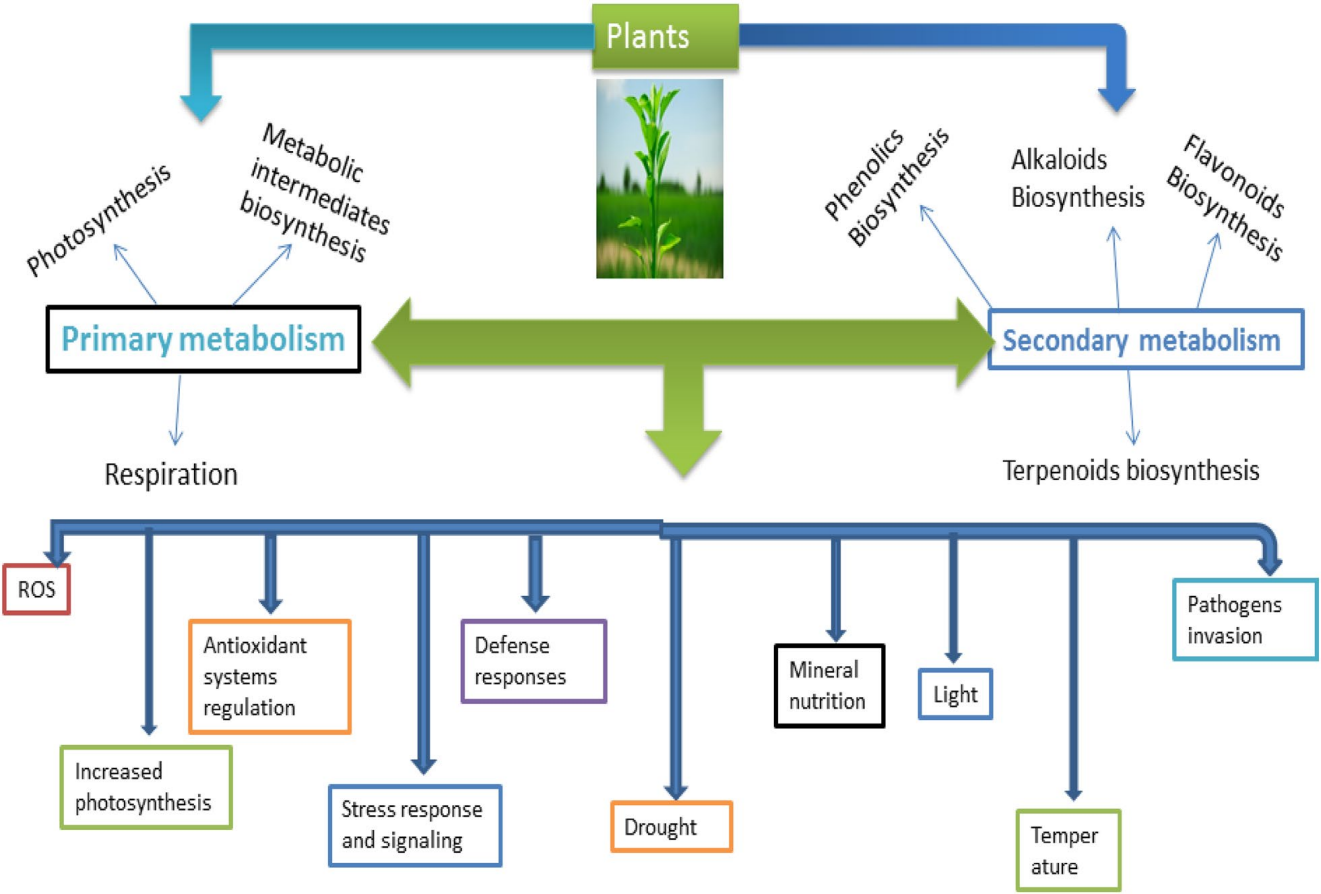
In their natural and in vitro growth conditions, plants encounter variety of stresses and biotic disturbances which leads to the initiation of stress and defense responses mediated by signaling processes and pathways involving repertoire of molecules to perform cellular functions essential for physiological processes. The physiological processes impact primary metabolism that provides biosynthetic intermediates for secondary metabolism, with concomitant effect on biomass and bioactive compounds biosynthesis. This generally depends on the species, genotype and cultivar as well as the stage of development and physiological state of the plant investigated

Literature reports in the past decades, had shown that during in vivo growth condition of plants, adverse environmental stress and climatic factors that includes drought, temperature extremes (freezing and heat), light irradiance, nutrients deficiency and soil contamination with high concentrations of ions (metals and salts) are main stressors that influence plant physiology (Fig. 1) with stimulatory effect on SM in crops and medicinal plants [6, 97–103]. Similarly, the conditions of in vitro culture imposes a combination of stress factors to cultured plant cells through pronounced change in cellular environment that may be in the form of wounding of excised tissues, plant growth regulators (PGRs), salt concentrations (low or high) and high or low artificial light levels that could generate stress effects. This may lead to the induction of SM pathways, depending on the physiological state of plant cells [10, 55, 104]. Hence, of all the in vitro techniques applied in PSMs production, elicitation—which is based on the principle of stress induction, is the most effective strategy for enhancing production of the metabolites through the use of biotic and abiotic elicitors that promote biosynthesis of the molecules when added into culture medium during cultivation of plant cells, tissues and organs [13, 38, 55, 57, 105]. Further, because in coping with the in vivo and in vitro stress challenges, plants have evolved efficient mechanisms for recognition and adaptation to the elicitation, it indeed influences plant physiology and biosynthesis of the metabolites. This may involve adjustments in photosynthetic rates, stomatal conductance and transpiration (in vivo), cell wall architecture, membrane systems, alterations in cell cycle and division rates (Fig. 1) with overall effect on general growth to fine-tune physiology and metabolism of bioactive compounds [106, 107]. It may involve expression or repression of gene regulatory network in response to the stress effect(s) [61–63] to confer tolerance at cellular levels by producing tolerance-associated molecules essential for regulation of signal transduction systems and stress responses [64, 68]. For example, production of flavonoids and cinnamic acid derivatives during drought-induced stress tolerance in cotton suggests their high efficiency in ROS scavenging [108] while isoprenes production due to heat-induced stress indicates their effective oxygen quenching antioxidant capacity in reed plants [109–111] Phenylamides are produced for efficient quenching of singlet oxygen radicals in plant cells during stress [112] while phenylamines accumulation in tobacco and bean due to abiotic stress suggests their antioxidant role [10, 113, 114]. Flavonoids, terpenoids, and volatile secondary metabolites provide color and scent properties to plants, which entails repellent and attraction effects on insects and herbivores, while toxins could be involved in plant-plant allelopathic effects [10, 115]. Generally, during both stress and defense response in plant cells, the fixed carbon through photosynthesis becomes allocated to SM with an overall effect on growth inhibition (Fig. 1), and synergistic effect may be encountered in some plant systems [20, 116]. For instance, biosynthesis of phenylpropanoid SMs in tobacco showed regulated control by carbon–nitrogen status and the production was confirmed by gene expression studies [117]. Combined effects of pest with the abiotic stress promoted production of SMs in cotton [118] while combination of wounding with water-induced stresses showed synergistic action in the production of phenylpropanoid SMs in carrot [119]. However, this depends on the sampled plant species, cultivation season, genotype and cultivar investigated [7, 8, 101, 120, 121]. The differences can be ascribed to the cellular receptor specificity, subcellular localization of ROS production, specific and regulation of MAPK activities, existence of differences in the activation of genes and product of their expression among plant species, genotypes and cultivars, and in relations to the inducing environmental factors [24, 122, 123].

## Stress and defensive response initiators in relations to the plant secondary metabolism

Defense response mechanism(s) involve specific modification in the state of metabolic gene expression network which effect protein synthesis to modulate associated primary and SM pathways. The variety of biotic and abiotic stresses encountered by plants during in vitro and in vivo growth conditions impact physiological processes [124] with triggering effect on biosynthesis of PSMs (Fig. 1; Tables 1 and 2). In the past recent decades, reported studies on the plant defense response(s) system(s) in relations to the SM induction are on hypersensitive response, PR synthesis, systemic acquired resistance and production of phytoalexins [20, 21, 24, 26]. Abiotic stress factors have received more comprehensive investigations due to the ease at which their effect on plant physiology and SM can be studied when compared to the biotic, although few studies have explored their combinations [11, 101, 124]. For instance, exposure to the biotic and abiotic stresses (in the form of temperature, nutrition, cutting, light, PGRs and water) and during developmental processes, in relations to their stimulatory effect on camptothecin (CPT) biosynthesis were comprehensively investigated in *Camptotheca acuminata* [125–128]. The role of nicotine and caffeine as strong insecticides in tobacco and coffee plants have also been studied through their accumulation or secretion in cells [72, 129]. Production of anthocyanins can be stimulated by treating the plant sources with sugar and nutrients deficiency, pathogen attack, wounding, high light intensity, ultraviolet and blue light radiations [6, 130]. Seasonal variation influenced production of sesquiterpenes, lactones, and phenolics in the leaves and stem of *Tithonia diversifolia* and correlation with the amount of rainfall and temperature changes were established [131]. Extensively studied phenyl propanoids on their biological activities and biosynthesis have been reported to be involved in plant defense responses, in addition to structural components formation in plants (e.g. lignin synthesis essential for cell wall formation) and abiotic stress tolerance [82, 132]. The above metabolites and many reported others could serve as markers of stress or defense response function been employed by a plant species, variety or cultivar under a given growth condition, and could be a base for search of novel bioactive compounds produced by plant system(s) in response to changing conditions of the in vitro or in vivo growth.

### Nutrition

In the condition of plant growth, exposure of cells, tissues or organs to nutritional stress may result in the accumulation of osmo-protectants to stabilize photosystem II complex, enzymes, and proteins structure, maintain membrane integrity and ROS scavenging [11] with possible marked effect on biomass and SMs production [47, 124]. Recent studies have shown that mineral nutrients may enhance or suppress growth, and biomass production exerts effect on SM modulated by growth condition and environmental factors, depending on the status (high or low concentration) and species or genotype physiology and developmental stage of plant studied [8, 124, 133]. For instance, micro nutrients availability may impact production of bioactive compounds through effect(s) on biosynthetic pathways as activators of enzymes while macro nutrients such as carbon and nitrogen have particular relevance for biomass and SMs biosynthesis [24]. Nitrogen may influence growth and development through primary and SM, and a link could be established between the two metabolic pathways through phenylalanine ammonia lyase (PAL), explaining the influence on the production of flavonoids in plants due to higher PAL activity [134–136]. Phosphorus, a part of energy-rich molecules such as ADP and ATP is involved in primary metabolism in plants, and its deficiency could induce anthocyanins production accompanied with decreased development [47, 137]. Magnesium plays several functions in plants that include ATP synthesis, CO_2_ fixation, chlorophyll formation and assimilation of photosynthetic products, generation of ROS and photooxidation of leaf tissues, and as part of chlorophyll structure. Its deficiency may result in an increased production and accumulation of active oxygen species in plant cells that may effect production of carotenoids [47, 138]. For phytochemicals whose production in plants is influenced by sulfur (S) availability, fertilization in deficient ones enhances production while stimulatory effect could not be found in S-sufficient ones [139]. Specific micro nutrients that play cellular redox function that include Cu, Fe, Mo, and Mn may serve as factors for some PSMs biosynthetic pathways [24, 25]. For instance, Cu plays important roles in many oxygenases and oxidases that play an essential role in secondary metabolic pathways, as in putrescine and cadaverine biosynthesis where it inhibited the activity of diamine oxidase [24, 140]. Hence, optimizing mineral nutrition of medicinal plants during hydroponics, aeroponics and in vitro cell culture pose considerable challenge given the supra-optimal effect of nutrients supply on growth and SMs production [24, 47, 124, 141]. For instance, application of aeroponic and hydroponic culture systems of *Withania somnifera* differentially influenced biomass and Withaferin A production but, hydroponics was the most effective [142]. However, differential production of the PSMs can be encountered in the hydroponic growth condition, based on plant species, variety, genotype and culture conditions [47, 124]. For instance, in a recent study using shoot cultures of *H. perforatum* grown in non-aerated liquid medium systems, higher shoot growth, phenolic compounds, and hypericin production were achieved under total and partial immersion over paper bridge support cultivation system [143].

In many recent literature reports, the most employed strategy in studying the effect(s) of nutrition to PSM involves experiment(s) on physiological changes induced by in vivo growth condition through evaluation of metabolites profile in response to supra or suboptimal concentration of nutrients, and involves evaluating their influence on growth, development, and biosynthesis of the PSMs [47, 124, 144]. This offers a “snap shot” about the effect of nutritional priming to plant physiology that involves stress or defense response function. For example, Nitrogen, Phosphate, Potassium, and Sulfur-induced stresses influenced the biosynthesis of phenylpropanoids and phenolics in several plant species studied [6, 97, 133, 144, 145]. Production of CPT in the leaves of *Camptotheca acuminata* seedlings showed variation in response to different forms of nitrogen sources [128]. Significant higher phenolic compounds were accumulated by the leaves of *Olea europaea* trees subjected to boron deficiency when compared to the semi-hydroponic cultures [146]. Calcium, a ubiquitous signaling molecule involved in many signal transduction pathways in plant cells had been shown to become elevated in cellular levels in response to light, salinity, drought and cold stresses of the in vivo growth conditions [147] and influence in vitro morphogenesis [148, 149]. Variation in nitrogen source and ratio showed influence on biomass and azadirachtin production in cell suspension cultures of neem variety with up to 1.5-fold enhanced production in the extracellular, whereas reduction in phosphate level in the culture medium reduced the intracellular levels [150]. The omission of nitrate in the culture medium of *Chrysanthemum cinerariaefolium* during a second phase of culture induced increase in Pyrethrin production by twofold [151] while root cultures of *Morus alba* grown in medium that contained lower NH_4_^+^/NO_3_ ration resulted in greater production of rutin compared to the higher [152]. In *Catharanthus roseous*, salinity stress along with the nitrogen sources influenced antioxidants activity and indole alkaloids production [153]. The above examples have shown the complex and differential cross-talk in secondary metabolic pathways signaling associated with stress and defense response function influenced by nutritional state of plants during in vivo and in vitro growth conditions. Nutritional priming-based in vivo and in vitro experimental approaches were the most employed in influencing gene expression of the metabolic pathways in the studies, and effect metabolites biosynthesis profile through enhanced SM with beneficial effect on resource-use efficiency of the plants. In many of the reported studies, higher production of the secondary compounds contributed in preventing damage caused by the production of free-radicals associated with nutrition-based stress and defense response function, evidenced by changes in biochemical profile of the analyzed tissues. Molecular biology tools and metabolites profile techniques were also employed in investigating the metabolic expression of the plants in response to stress and defensive response functions induced by the nutrition. Macro and micro nutrients influenced SM, and optimal nutrition can enable plants cope with nutritional stress situations induced by biotic and abiotic factors, as well as during defensive signaling function that have influence on yield of the metabolites. A nutritional state that maintains suitable C/N balance combined with appropriate growth condition of plants that includes light condition and intensity, plant physiology, genotype and age are key determinants for accumulation of PSMs under nutrition-influenced stress and defense response function.

### Drought

Drought, typically associated with high photoinhibition and temperature stresses, is among abiotic stresses that exert great affect on plant growth and development. It occurs due to water deficiency when their availability become reduced to critical levels accompanied by high solar radiation and temperatures [6, 154, 155]. This may cause many changes in physiology and biochemistry of plants, including arrest of cell growth and photosynthesis with an enhanced respiration [156]. Thus, may affect biosynthetic pathways for the production of PSMs through provision of precursors or intermediates from the primary metabolism. In many recent reports, it had been shown that exposure of plants to the drought promoted higher production of various classes of SMs that include terpenes, complex phenols, and alkaloids during in vitro and in vivo growth through the induction of ionic or osmotic stress [32, 46, 157–159]. However, in most of the reported cases, the increase was accompanied by decreased biomass production [27, 46]. Such cases are exemplified in *Hypericum brasilience* and *Pisum sativum* where concentration and amount of phenolic compounds biosynthesized were drastically enhanced when the plants were grown under drought stress in comparison to the control [160, 161]. In a similar report on the biosynthesis of terpenes in *Salvia officinalis*, higher biomass loss was accompanied by elevated levels in the production of monoterpenes [162]. Oxidative stress caused by the drought promoted biosynthesis of flavonoids [163], and was implicated in protecting plants grown in soils rich in toxic metals such as aluminum [25, 163–169] while production of shikonin, tocopherol and digitoxin in plant cell cultures were influenced by treatment of the producing plants with Ca^2+^ and its chloride, Fe^2+^, MnSO_4_ and cadmium [170–173] in differential manner. Drought-induced stress enhanced production of SMs in the leaves of willow plants [174] while decreased production of saponins was encountered in *Chenopodium quinoa* when grown under low water deficit [175].

The drought condition can be mimicked in the in vitro plant cell culture by media manipulation, encapsulation-dehydration methods or cryopreservation approaches, and both have proved efficient in promoting SM in many plant systems [176, 177]. In many recent studies it had been shown that composition of the culture medium that includes nutrients, carbon sources and osmotic stabilizers can be manipulated to create in vitro drought conditions, with effect on metabolic processes that may lead to biomass and SMs accumulation [101, 177, 178]. For instance, media nutrients manipulation influenced biomass and CPT production in *Nothapodytes nimmoniana* [179, 180] and *Ophiorrhiza mungos* [181]. The strength of the culture medium nutrients influenced phenolic compounds profile of *Bellis perennis* calli through the induction of antioxidant system due to increased stress condition [182]. AgNO_3_ or CdCl_2_ stress enhanced production of tropane alkaloids (hyoscyamine and scopolamine) in treated hairy root cultures of *Brugmansia candida* [105].

Cryopreservation, a technique by which plant cells, tissues, organs and extracellular matrix or other biological constructs liable to be damaged due to unregulated chemical kinetics becomes preserved through cooling at very low temperatures has proved efficient in the conservation of many herbaceous and woody plants for the production of secondary compounds [183–186]. Exposure of the plant cells, tissue or organ to cryopreservation may induce changes that include desiccation, osmotic injury and low temperature-induced stresses [186]. The technique was found ineffective in the production of phenols but, enhanced the biosynthesis of flavonoids in the cryopreserved and regenerated species of *Hypericum* [184, 186] while *Rhodiola crenulata* calli showed enhanced survival when pre-treated with 0.1 µM melatonin [187]. When *Taxus chinensis* cell suspension culture were cryo-preserved for up to 30 days, good recovery of the cultures with retained stability in paclitaxel biosynthetic capacity was observed in comparison to cultures maintained through regular subculture [188]. On the other hand, cryopreservation of *H. tetrapterum* shoot cultures did not alter biosynthesis of phenol but enhanced the yield of flavonoids, with effect on growth, biochemical and cellular processes [184, 186]. The shoot tip of *H. perforatum* cryopreserved showed genetic stability with sustained production of hypericin after recovery of meristems, and at levels similar to the unfrozen control [189, 190]. Vitrification and encapsulation-dehydration techniques of cryopreservation applied to the *Dioscorea deltoidea* resulted in high frequency regeneration of plantlets with stability in diosgenin content as the control [191]. The use of minimal growth conservation coupled with genomic DNA methylation manipulation sustained paclitaxel production in the cell cultures of *T. media* that showed decreased yield upon repeated subculture [192]. Expression of foreign genes and enzymatic activity of SMs biosynthesis were maintained after cryopreservation of *Papaver somniferum* cell cultures [193]. Generally, stress and defense-associated SMs biosynthetic response varies with the state of plant growth, and effect of drought on SMs profile could be associated with biomass accumulation through changes in growth and developmental physiology. Thus a shift between vegetative and generative plant growth physiology may be encountered, with impact on source-sink metabolic state of a plant during in vivo or in vitro growth and an overall effect on metabolites profile. Approaches that enhance metabolites profile and concentration through elevated biosynthesis of the compounds could compensate for lower yield encountered with most plant species. In recent years, application of irrigation systems manipulation in the in vivo growth [124], and PGRs alone or in combination with signaling molecules manipulation during in vitro culture have found application in enhancing SM of plants. However, the impact and efficiency of enhancing productivity of the PSM using the approaches varies with species and experimental system employed [101], given the varied impact they have on primary metabolism pathways and developmental processes that are still difficult to evaluate at cellular level in plants. In this context, evaluating the growth characteristic and metabolite profile is an alternative way for deducing the impact of drought to SM in plants, which is the current approach employed in most of the reported literature. Although drought negatively impact plant growth through biomass production in most of the reported experimental plant systems, it indeed enhances SM. Thus could be an explanation to the higher natural product profile and yield encountered with plants grown in tropics or in vitro cultures subjected to the elicitation of biosynthesis using biotic or abiotic stressors.

### Temperature

Among the harmful abiotic stresses that impact plants survival in temperate climate is low temperature, and species adapted to the condition adjusts metabolic processes to increase levels of cryo-protectants essential for tolerance during the autumn [180]. Varying temperatures of the in vivo and in vitro condition under which plants are grown impact metabolic processes and ontogeny, and higher may induce premature senescence of leaves, with impact on PSM [5]. For instance, temperatures and phenological stage impacted SMs production in *Rhodiola rosea* clones [194] and elevated levels combined with heavy metal stress promoted SM with synergistic action implicated [195]. Light and temperatures showed synergistic action on the production of SMs in the callus cultures of *Helicteres isora* [196]. Production of polyamines and subsequent formation of phenylamides had been shown to occur in bean and tobacco when subjected to heat shock and water stress, with the phenylamides ascribed ROS-scavenging function during the stress [10, 113, 114]. Similarly, thermal treatments slightly decreased the production of carotenoids produced by the Brassicaceae members [5] while elevated levels promoted leaf senescence and root SM in *Panax quinquefolius* [197]. Cold stress promoted the production of phenolics and their subsequent incorporation in plant cell wall as suberin or lignin [198] while tree adaptation to the cold climate was associated with the production of chlorogenic acid at high levels [199]. Sometimes variations in temperatures may have multiple effects on the expression of metabolic processes involved in the production of SMs through regulation, permeability and intracellular reactions rate in plant cell, tissue and organs by influencing physiology and metabolism of the plants. This may have marked effect on growth, cytodifferentiation and production of the molecules [5, 35]. For example, low and higher temperatures showed influence on SMs production in somatic embryos of *Eleutherococcus senticosus* through provoking oxidative stress that was more prominent at higher temperature over the lower [200]. The temperatures along with light quality influenced the production of ginsenosides in hairy root culture of *Panax ginseng* [201] while cell cultures of *Melastoma malabathricum* incubated at low temperatures produced higher biomass and anthocyanins than those grown under the higher [202]. Changes in temperatures of incubation influenced SMs production in the callus cultures of *Brassica napus* through induction of oxidative stress, as confirmed by antioxidant enzymes activity [203]. It influenced accumulation of flavolignans in the hairy root cultures of *Silybum marianum,* with acid pH proving the most efficient when combined with the treatments [204]. The growth of hairy root cultures of *Stevia rebaudiana* was affected by increase in temperature of incubation conditions while the increment enhanced production of stevioside up to certain levels [205]. Hence, each plant species, cultivar or genotype have specific optimal temperature ranges for physiological functions that includes biosynthesis of the SMs, and deviation from those ranges could impact biomass and biosynthesis of the SMs. Thus, variations in yield of the metabolites could be encountered across seasons and regions of the world during in vivo growth of a specific plant species, cultivar or genotype, and could in turn be mimicked in the in vitro cultures by media manipulating/cultural conditions, the basis through which many in vitro-based SM enhancement strategies have been developed.

### Light

Plant species or even cultivars vary in their physiological response to light condition exposure in the form of photoperiod or short duration(s) associated with SMs production during in vivo and in vitro growth conditions [101]. Solar radiations reaching the earth surface encompasses UV-A, UV-B, photosynthetic active and infrared but, only small proportion of UV-B that forms the most energetic component of day light spectrum is used by plants for growth and development, depending on the exposure wavelength and interaction with environmental signals [206]. The light is also regarded among limiting factors that affect growth and development in plants during both in vivo and in vitro conditions, and can affect SMs production, depending on species or genotype, stage of development, light type, and exposure duration [101, 207]. For example, accumulation of SMs under different temperatures, light intensities, and phonological cycle during greenhouse growth of *H. perforatum* showed variability for each of the specific compounds evaluated [208]. Phenotypic plasticity associated with the light and nutrients condition influenced biomass and iridoid glycosides accumulation in *Plantago lanceolata* offsprings [209]. Its quality influenced growth and flavonoids production in *Hyptis marrubioides* seedlings cultivated in vitro with red light as the most effective for plant growth and leaves production while blue and white for the promotion of rutin accumulation [210]. Exposure of American ginseng plants to the sunlight at longer duration promoted higher ginsenoside production in roots than those exposed to shorter period of direct sunlight treatments [211]. In *Catharanthus roseus*, exposure to UV-B light significantly impacted biosynthesis of vincristine and vinblastine, which are effective anti-lymphoma and leukemia drugs currently in use [212]. Intensity and duration of the light exposure influenced biomass and CPT content yield of *C. acuminata* seedlings, and was confirmed by the expression of genes that participate in its biosynthesis [213]. In the same species, enhanced expression of Tryptophan decarboxylase 1 (TDC1) was regulated by chemical defense systems while TDC2 acts as an integral part of the process induced during challenge imposed by a pathogen [214]. Light conditions showed substantial effect on SM in the shoot cultures of *Scutellaria lateriflora* with blue light been the most effective, and relationship with PGRs was established [215]. Its various spectral levels influenced caulogenesis, biomass and SMs production, and were dependent on the stage of calli growth [216]. Exposure of *Peucedanum japonicum* callus cultures to the different light spectra provided by light-emitting diode sources showed their influence on calli proliferation and the number of somatic embryos differentiated, as well as SMs biosynthesis, with red and blue light as the most effective [217]. Light and dark conditions of incubation showed substantial effect on biomass and SMs production, based on the culture duration of *Artemisia absinthium* cell suspension cultures [218]. It stimulated gingerol and zingiberene production in *Zingiber officinale* callus cultures [219] while the type influenced CPT biosynthesis in *C. acuminata* seedlings [220, 221]. Biosynthesis of artemisinin in the hairy root cultures of *Artemisia annua* was influenced by the light irradiation [222], and white light affected the production of taxol and baccatin III in the cell cultures of *Taxus cuspidata* [223]. Elicitation of *Eurycoma longifolia* calli with UV radiation resulted in the production of compact calli with elevated levels of alkaloids biosynthesis over the control [224]. It is apparent that the influence of light on plant growth and SM is multi-faceted and dependent on the species investigated [101] during in vivo or in vitro growth stage(s), and physiological state of tissue or organ evaluated, more importantly spectral level of the light source.

## Secondary metabolites production as salinity tolerance mechanism in plants

Anthropogenic activities that promote soil salinization are enlarging the percentage of worlds salinized land mass and have impact on the survival of medicinal plants as well as availability of the bioactive compounds they produce [1]. Genotypic plasticity of the plants in changing and challenging environment of in vitro and in vivo saline growth conditions enable them produce repertoire of SMs essential for survival under the physiological perturbation, and varies with species, genotype and salinity stress levels. Physiological, biochemical, morphological and biosynthesis impact of the salinity on plant natural products profile through induction of oxidative stress and defense response pathways involves production of ROS which plays essential role in altering PSM in medicinal plants [225–227], and is increasingly understood due to advances in application of molecular profiling and finger printing techniques in many plants and natural products they produce [132]. Considerable progresses have been made on the identification and characterization of different salt stress-induced responses associated with PSMs production and their mechanism of accumulation in number of medicinal and crop plants [1, 132, 228]. Salinity-induced stress leading to the secondary metabolic pathways induction may also be initiated by drought that causes accumulation of solutes at higher levels through osmotic adjustment. At initial stages of the salinity-induced stress, ability of roots to absorb water becomes drastically reduced. This may lead to loss of water due to osmotic stress mediated by accumulation of salts at higher levels in plant and soil [226, 229, 230]. Consequently, physiological changes that interrupt membrane functional stability, redox homeostasis and nutrients balance becomes affected, with overall effect on primary metabolism that provide precursors to SM pathways and stomatal function [132, 226, 231, 232] that are connected to metabolic changes associated with PSMs biosynthesis which may sometimes involve circadian rhythm response [231]. Tolerance to the salinity-induced stress is the ability of a plant species to sustain cellular metabolic processes through systemic adjustment in physiological processes [225]. In such plants, physiological changes that include salt exclusion and sequestration, tolerance to accumulated ions and restricted loss in K^+^, water homeostasis and osmotic adjustment control, along with growth and enlargement modification through biochemical expression are commonly encountered [232–235]. In the case of extremophiles which are adapted to the saline growth conditions, they pre-adapt by increasing the levels of SMs biosynthesis induced by salinity stress, and decrease in their production and salt-stress levels negatively affects physiological processes [234]. Thus, determining impact of salinity on plant physiology involves studying many physiological variables and their interactions over time.

Over the past recent decades, physiological and molecular effect of salinity-induced stress on growth and production of important PSMs in crop species have been investigated. In the case of medicinal plants, the information is still lacking, especially variable stress and defense responses associated with their production [103, 225, 226, 228, 236–239]. Significant number of PSMs, classified as terpenoids and steroids, phenolics and flavonoids and alkaloids have been reported to be produced, involved or become activated in cellular stress and defense response function influenced by saline condition of plant growth physiology [228, 240, 241]. For example, production of aromatic compounds (e.g., alkaloids, isoprenoids and phenols) and phenylpropanoids-derived compounds (e.g., tannins, flavonoids and hydroxycinnamate esters) at higher levels is regarded to be mediated by salinity stress and free-radical scavengers which constitutes an adaptation to the condition in SMs-producing plants [228, 236]. Important physiological changes that determines survival of a plant species and production of the metabolites under the saline growth conditions of the in vitro or in vivo includes osmotic adjustment that involves production and accumulation of cellular osmolytes (polyols, proline, sugar alcohols, pinitol, glucosinolates and glycine betaine, etc.) and soluble sugars (glutamate, sorbitol, mannitol, oligosaccharides, fructans and sucrose, etc.) [225, 228, 242, 243]. Biochemical markers for the salinity stress tolerance includes accumulation of cellular osmolytes (e.g. polyamines, proline, soluble sugars and glycine betaine), partly for the role they play in maintaining stability of membrane and other cellular structures [225, 244]. High production or expression of antioxidant system (enzymatic and non-enzymatic) to sustain cellular function crucial for physiological stability of plants under the saline growth condition could also be used as marker of salinity stress-induced PSM. Phenolics are produced by many plant species for protection against biotic or abiotic stress growth condition(s) and their accumulation correlates with antioxidant capacity of plants in number of species [245–247]. However, the effect of salinity on PSM induction, with respect to expression of the above changes have been evaluated in most cases in medicinal plant systems through studying changes in carbon and oxidative metabolism, nutrition and ionic accumulation which translates into decreased growth and development, and impair physiological processes associated with PSMs production [225]. The effect could be short or long-term; reduction in water uptake, osmotic stress and lowering in external water potential are regarded as short-term while ions-induced toxicity due to inability to properly compartmentalize ions are long-term effects [226]. Thus, exposure to salinity stress may serve as elicitor to SM to serve protective role on cells from oxidative injury that may be caused by accumulation of ions at cellular and subcellular levels, thereby reduce its toxicity effect [248]. For instance, polyols that includes sorbitol and mannitol, glycinebetaine, fructans and trehalose sugars and proline, among others, play an osmolyte role in cells through alleviating stress arising due to exposure to the salinity stress in growth condition of plants by elevating their levels of production and generation of higher or “over supply” of reducing equivalents [249]. Their production at higher levels under the condition may be induced by alteration(s) in cellular ion uptake, transport and balance, hormone and antioxidant metabolism, osmoregulation and other stress signaling critical for adaptation to the salinity stress [132]. For halophytes that spend substantial part of their life cycle under salinity stress—at least 200 mM NaCl (Flowers et al. 2008), cellular osmotic pressure is “high enough” to enable them sufficient water uptake in the saline environment, and at the same time, produce secondary metabolites under such physiological condition through variety of mechanisms [226]. In the halophytes, salinity levels may cause cellular dehydration through ions accumulation (mainly Na^+^ and Cl^−^) that in turn results in osmotic stress induction arising from water removal from the cytoplasm, with resultant effect on reduction in vacuolar and cytosolic volumes as well as PSMs biosynthesis [250]. Leaf cells of these plants are able to remove Na^+^ and Cl^−^ from the cytosol, followed by their sequestration in vacuole through displacement of nutrient ions that includes Ca^++^, K^+^ and nitrate which negative affect plant survival [237, 251]. According to Briens and Larher [252], halophytes could be classified into three physiotypes based on the compounds they accumulate when grown in saline condition. (1) Those that produce soluble carbohydrates and/or polyols at higher levels with a low water-soluble nitrogenous compounds, (2) Those that accumulate high-level water soluble nitrogenous compounds than non-structural carbohydrates, and (3) Plants accumulating both nitrogenous and carbohydrate solutes, with the first been most quantitatively dominant in the reported literature. Even among halophytes and in accordance with Shelford’s law of tolerance, variation in tolerance to the salinity stress levels are encountered due to differences in tolerance range for a given physiological function of a plant species, variety or cultivar, determined by genetic make up [253]. For instance, in calli of *Solanum nigrum*, exposure to the salinity stress levels resulted in correlated enhancement on production of solasodine and proline for its tolerance [254]. In *Sesuvium portulacastrum*, exposure to salinity of 800 mM NaCl impaired physiological processes through production of SMs and other biochemical changes, with strong antioxidant capacity playing vital role under the extreme saline condition for its survival [255] that in the case of other species could strongly impede physiological processes or death of plants [226]. In number of medicinal plants, drought-induced salinity had been implicated in enhanced SM through alterations in plant growth physiology in differential manner, based on plant species, genotype and cultivar investigated [256–258]. Thus, accession-dependent variation in production of the PSMs and antioxidant capacity during exposure to the salinity stress of in vivo or in vitro growth condition of plants could be attributed to differential response to sustainable growth condition [228, 256, 259]. For instance, Szabó et al. [260] examined the effect of 5 days drought on alkaloids production in *Papaver somniferum* with narkotine, codeine and morphine detection peak been higher after short exposure duration, possibly due to the influence of salinity on cellular function through metabolic biochemical pathways essential in maintaining cellular stability similarly also reported in *Catharanthus roseus* [261]. Exposure to differential salinity stress levels resulted in stimulatory effect on biosynthesis of oleuropein and phenols at higher levels in leaves over other tissues of four *Olea europaea* cultivars in differential manner [262]. Tissue-dependent enhancement in the production of polyphenols in response to the salinity stress have been reported in many plant species [255, 263]. In summary, literature reported studies about effect of salinity on SMs production using the approach of ecological metabolomics and in vitro culture systems have helped in elucidating differences between salt-sensitive and tolerant species, as well as their diversity pattern in botanical kingdom, and have application in development of crops capable of adapting to the condition, particularly in tropical areas where the levels of soil salinization is on the increase due to climate change and anthropogenic activities. Over the past recent decades, primary metabolism-based biochemical changes in expression levels are main markers used in generating data associated with PSMs production in most reported experiment results. This have helped in furthering knowledge about plantsˈ salt-stress physiological response and adaptation over time in many species, cultivars and genotypes. Thus, knowledge on plant salt-tolerance and signaling networks is beneficial in developing salt-tolerant plants through metabolomic, genomic, transcriptomic and proteomic approaches, particularly when performed in complementary and integrative manner with biosynthetic pathways elucidation. This have also been of significant benefit in understanding salinity stress-induced complex responses at molecular, cellular and whole plant physiological levels associated with SMs production in different plants species. However, synergistic influence of salinity-induced stress with other environmental growth conditions on SMs production in medicinal plants are poorly understood. Understanding salinity-associated changes in receptors, sensors and signal transduction signaling systems, the molecules involved in long-distance transmembrane ions transport will be key to elucidating the intra- and intercellular molecular interactions associated with plantsˈ salinity tolerance responses, as well as genetic engineering for development of salt-tolerant crop and medicinal plants.

## Application of global metabolic analysis using LC–MS in deciphering abiotic stress tolerance mechanisms associated with plant secondary metabolites production

It is generally accepted that plant metabolome—the complete set of low molecular metabolites produced in cells, tissues and organs of a plant species, cultivar or genotype at set time and under certain condition of growth and development, modulates processes at macro molecular levels through providing integrated functional view of plant via application of many analysis techniques [264–266]. This has been made possible by the application of metabolomic analysis which have facilitated global profiling and characterization of large number of SMs produced by many plant species under variety of environmental growth conditions [83, 267, 268], with abiotic stress tolerance mechanism of crop and medicinal plants been the most investigated [60, 265, 266, 269]. Metabolomics-based techniques such as phytometabolomics, lipidomics and sensomics, among others, have facilitated the unraveling of many metabolic pathways, understanding of the biosynthesis of metabolites having differential bioactivity that becomes up or down-regulated in expression due to stressful growth condition of plants and signal transduction transmission in cells. The metabolomic analysis of a plant samples can performed in situ [264, 265] for a targeted and non-targeted metabolites characterization [4, 249, 268]. Recent developments in genome selection have revealed many encoded potentials of plant species on the production of diverse bioactive compounds of therapeutic use, particularly those produced at low levels and difficult to detect by standard methods [268, 270–272]. In this context, deciphering gene function under the stress and defense responses functions via biochemical kinetics essential for SMs production and consequential instrumental profiling needs holistic approach, so as to effectively gain good understanding of the impact of environment on PSM in challenging and changing environment of plantsˈ growth conditions [268, 272, 273]. This becomes necessary due to similarities in cellular and biochemical physiological changes that may be induced by change in gene expression and SM when a medicinal or crop plant species is subjected to similar or varied environmental stress levels [268, 274, 275], depending on the stage of development, plant species, genotype or cultivar investigated [4, 242, 266, 273, 275]. Despite the wide application of genomic, transcriptomic and proteomic approaches in deciphering molecular mechanisms of the PSMs biosynthesis and accumulation in several of the crop and medicinal plant species, information(s) about their production is still limited [60, 167, 237, 267–269, 272]. Thus, understanding the molecular mechanism of their production will be key to exploring maximal application as pharmaceuticals or herbal drugs, fragrances, flavors or spices due to the production in most cases in rare or endangered plant species, the high cost of the fine chemicals and low production, in addition to limitations in genomic information essential for the analysis.

Metabolites analysis could be performed based on mass spectrometry (MS) that involves recognition and quantification of plant secondary compound(s) in sample. It could be carried out in precise way to gain information about nature of a compound produced via ionization using positive and negative ion mode [276] that may involve the use of time-of-flight (TOF) and quadruple or ion trap analyzers [277]. The MS system may be hyphenated to chromatographic techniques but, the method chosen for an ionization and type of analyzer used in mass spectrometer during the chosen analysis determines detection efficiency of a system used. In the course of MS analysis, ionized molecules are measured and value of mass to charge ration of the produced ions from a sample are separated either in analyzer at accuracy of one mass unit, high and low-resolution mass spectra or to the 4th decimal point. A high-resolution mass analyzer enables researcher make conclusion(s) on elemental composition of ions that got detected in mass spectra, which is then beneficial in studies aimed at characterizing structure of a bioactive compound [269]. Thus, in the case of protonated or deprotonated molecule(s), molecular mass and elemental composition of a molecule(s) in *m/z* values could be estimated. The product ion or fragments registered in the MS spectra provides additional information or data about structure of an analyzed compound, and degree of unambiguous identity is dependent on the MS system employed. Among the highly used MS interfaces employed in the analysis of PSMs based on atmospheric pressure ionization strategies include atmospheric pressure photo-ionization, electrospray ionization, and atmospheric pressure ionization techniques. Overall, the MS system separates metabolites based on *m*/*z* ratio of their ions in sample, and comprises of an ionization chamber for ionization of component molecules, mass analyzer that separates ions by their *m*/*z* through application of electromagnetic fields and detector that record *m*/*z*. In the case of liquid chromatography (LC)-MS, capability of physical separation using LC is coupled with mass detection and analysis capacity of MS when analyzing PSMs in sample. In such most recent and advanced technique in use for plant metabolomics analysis, which is based on the principle of mobile and stationary phases, an interface that effectively channel separated components from an LC column (through pressured mobile phase) into MS ion source that analyze its components under vacuum operation are set up for the analysis. The solvent to be used for the LC–MS metabolomic analysis should (preferably) be dissolved in solvent similar to the high-performance liquid chromatography (HPLC) system eluent. Thus, the interface facilitates LC–MS transfer of maximum amount of an analyte by removing portion of mobile phase used, and at the same time preserve chemical identity of chromatographic analysis product without interfering with the efficiency of ionization and MS system vacuum efficiency. Because the library used for structural identification of a compounds in a sample using the LC–MS are less developed, the use of instrument-type-dependent mass spectra, fragmentation pattern of MS, retention time shift (based on LC column used) are met with challenges when comparing structure identification results for compound. LC–MS system has been among the most employed technique in stress-induced metabolome changes evaluation in plants [278], and among the most employed LC–MS technique is reverse phase column due to its ease in separating majority of PSMs, determined by column packing particle size and internal diameter among other characteristics. In ultra high pressure liquid chromatography (UPLC)-MS, chromatographic resolution is improved through reduction in diameter of column packing material [279].

Metabolic analysis can be targeted to a bioactive compound or untargeted; in the case of untargeted metabolomic analysis, it can be performed to profile or monitor quantitative and qualitative change in composition of a biological sample material that was not obtained from different environmental growth condition of plant or in plants subjected to variety of stresses [4]. In such analysis, sample preparation involves extracting biological material using appropriate water-organic or organic solvent(s), followed by MS analysis using high-resolution analyzers through direct sample infusion into ionization chamber or chromatographic separation before the MS analysis [280, 281]. For targeted analysis, the prepared material sampled could be enriched with certain but defined compounds, so as to achieve utmost sensitivity of phytochemicals present in a prepared biological material under analysis using MS analyzer. Because of the lower ionization competition that may exist between metabolites present in sample, dynamic range of the MS instrument and ion source should be adequately addressed for proper interpretation of data registered through the identification results and statistical data obtained. Further, critical in the metabolomic analysis of plant phytochemistry is the confidence in accuracy of compound identification, based on chemical analytical technique employed [282–284]. By using bioinformatic analysis, it is possible to make a good comparison of data obtained from different objects, so as to visualize its correlation with data obtained using other OMICS approaches [285]. Through the application of metabolome and genome wide studies, Kusano et al. [286] and Matsuda et al. [287] succeeded in characterising flavon glycosides and many bioactive compounds in rice, and highlighted the metabolome potentials in a single plant species when adequately studied using combination of techniques. Metabolomics data generation using MS integrated with genomics and transcriptomics have also helped in deciphering many biological processes in *Arabidopsis* [288, 289], highlighting the complexity in metabolic profile of a single species in real time. Through the use of MS-based metabolomics, possible biomarkers for assisted breeding of barley cultivars resistant to *Fusarium* head blight were successfully characterized, with flavonoids and phenylpropanoid metabolites as the highly expressed plant secondary compounds [290, 291]. MS techniques have also found application in imaging metabolomics for which the metabolites arrangement in cell or tissue could be deciphered. For example, recent advances in applications of this technique in microbial studies using the imaging MS have made it possible to measure interactions in microbial colonies that produce SMs [292, 293], and at the same time determine and visualize spatial distribution of these metabolites in the colonies analyzed. In a study on metabolic interaction between colonies of *Streptomyces coelicolor* and *Bacillus subtilis*, Yang et al. [294] applied MALDI-TOF-imaging MS and succeeded in characterizing chemical identity and spatial distribution of compounds produced by the interacting and individual colonies through metabolic interaction between colonies of the two species when grown in proximity on agar plates. Despite this and many more other MS applications, many challenges still exists. The use of biological and molecular structural techniques in characterizing metabolite markers when combined with metabolomics will prove of significant benefit in deciphering plant metabolic response to stress and defensive situations in the in vitro and in vivo growth conditions. However, challenges need to be effectively addressed for efficiency in metabolic profiling and analysis. Critical among them is the insufficiency of MS analysis in getting detailed identification (that may involve structural elucidation) of bioactive compound(s) in a plant sample but, sufficient information for annotation and identification of an individual compound could be gained [272]. Another difficulty is the interpretation of MS data recorded due to the differences in ionization efficiency of an analyzed biological compound composition, because of the differential physicochemical properties that may cause different efficiency in deprotonation or affinities in the course of electrospray or atmospheric pressure chemical ionizations applied [295] in an instrument used. Therefore, when selecting suitable MS technique to employ and device for plant sample analysis, ionization source and detector to employ along with devise sensitivity should be put into consideration, due to their influence on signals detection for each sample run in an MS system.

## Conclusion and prospects

The influence of stress and defense responses to plant physiology associated with the biosynthesis of PSMs is multi-faceted, and increasing evidences from recent literature reports suggest the crucial role played by stress signal transduction system in their production. Ample number of the studies (e.g. Tables 1 and 2) have shown that both stress and defense responses are involved in SM in plants, although stress is the most investigated and understood. Because the responses are induced at subcellular level, their study is challenging. Thus, the use of biochemical and metabolic markers remains the most employed approach in drawing inferences on the impact of the stress and defense response to biomass and SM in the growth condition of plants in most of the reported studies using tissue or organs evaluated, rarely with whole plant system or cellular levels. During both responses, specific and non-specific reactions that permit adjustment of resource utilization by the plants from primary metabolism (Fig. 1) may occur, with possible impact on biomass and SMs production. The responses are dependent on the metabolic capacity of the studied plant determined by the genetic background, depending on the genus, species, genotype, and cultivar investigated, environmental factors and developmental stage. The physiological state also determines the expression of metabolic pathways for their production under the growth condition(s) involving variety of signature-markers expression that facilitates systemic signal transduction pathways adjustment in the in vivo and in vitro conditions. Influence of the responses in relations to the spatial and temporal changes in the production of PSMs in response to the signal transduction systems involved across species, genotype and cultivars still need to be studied. Exploring the physiological, metabolic status of plants in response to the stress and during defensive stimuli could provide a rationale for application in plant cell culture and metabolic engineering in the production of the high-value PSMs via application of next generation sequencing technologies and approaches.

## Data Availability

Not applicable.

## Abbreviations

SMs: secondary metabolites
PSMs: plant secondary metabolites
SM: secondary metabolism
PSM: plant secondary metabolism
ROS: reactive oxygen species
ABC: ATP-binding cassette
PAL: phenylalanine ammonia lyase
CPT: camptothecin
TDC1: tryptophan decarboxylase 1
MS: mass spectrometry
TOF: time-of-flight
LC: liquid chromatography
LC-MS: liquid chromatography-mass spectrometry
HPLC: high-performance liquid chromatography
UPLC: ultra high pressure liquid chromatography
PGRs: plant growth regulators
